# A convergent mixed methods to study registration on kidney transplantation waiting list refusal by women and men on dialysis in France

**DOI:** 10.1038/s41598-024-80775-7

**Published:** 2024-11-24

**Authors:** Latame Komla ADOLI, Arnaud CAMPEON, Valérie CHATELET, Cécile COUCHOUD, Thierry LOBBEDEZ, Florian BAYER, Elsa VABRET, Eric DAUGAS, Cécile VIGNEAU, Jean-Philippe JAIS, Sahar BAYAT-MAKOEI

**Affiliations:** 1 EHESP, CNRS, INSERM, Arènes – UMR 6051, RSMS – U1309 –, University Rennes, 35000 Rennes, France; 2 Arènes–UMR 6051, ISSAV, EHESP, CNRS, 35000 Rennes, France; 3 U1086 INSERM, Anticipe, Centre De Lutte Contre Le Cancer François Baclesse, Centre Universitaire Des Maladies Rénales, Caen, France; 4 Biomedecine Agency, Renal Epidemiology and Information Network (REIN) Registry, Saint-Denis-La-Plaine, France; 5 Inserm U1149, Université Paris Cité Assistance Publique-Hôpitaux De Paris Service De Néphrologie Hôpital Bichat, Paris, France; 6 CHU Rennes, INSERM, EHESP, IRSET (Institut de Recherche en Santé, Environnement et Travail) – UMR_S 1085, University Rennes, 35000 Rennes, France; 7 Unité de Biostatistique, Hôpital Necker-Enfants Malades, AP-HP; Institut Imagine, Université Paris-Cité, Paris, France

**Keywords:** Chronic kidney disease, Mixed methods, Gender/sex, Transplantation, Refusal, Epidemiology, Outcomes research

## Abstract

Not all patients on dialysis want to be registered on the kidney transplantation (KT) waiting list and undergo transplantation. The aim of this convergent mixed methods study was to determine the features of patients refusing to be registered on the KT waiting list and the reasons. Quantitative data on all 2017–2019 incident 18–85-year-old dialysis patients, eligible for KT, were extracted from the REIN registry in France. Qualitative data were collected through semi-structured interviews with patients on dialysis and nephrologists from the Bretagne, Île-de-France and Normandie French regions. The binary logistic regression method was used to identify factors/reasons associated with registration refusal and an inductive thematic analysis was performed on qualitative data. The quantitative analysis included data of 10,512 patients (mean age = 57.5 years). Among them, 860 (8.18%) refused to be registered on the KT waiting list. The multivariate analysis showed that women were 83% more likely to refuse registration compared with men. The qualitative analysis included 21 patients and 11 nephrologists. The integration of the results from the quantitative and qualitative analyses allowed identifying some factors associated with the registration refusal. Most of these factors converged across analyses. These included age, sex/gender, autonomy on dialysis and comorbidities. The integration of the results highlighted some divergence concerning sex/gender and autonomy and an area of expansion related to comorbidities. In conclusion, the patient age, sex/gender and comorbidities appear to play an important role in the refusal to be registered on the waiting list. Interventions focused on these factors might help to improve KT accessibility in France.

## Introduction

In the process leading to kidney transplantation (KT), registration on the waiting list is a key stage that involves medical considerations and the patient’s choice. Although KT is the best replacement treatment for eligible patients (i.e. patients without medical reasons for non-registration)^1,2^, not all patients seek to undergo this procedure^3^. In France, a patient is not registered on the waiting list due to medical contraindications, medical evaluation incompleteness, patient’s refusal, and other non-specific reasons. A French study found that after at least one year on dialysis, 14% of patients refused to be waitlisted, among whom 50% were women, although they represented only 41% of the studied population^4^. Similarly, a study in Slovenia showed that 14% of patients on dialysis refused KT^5^. An American study on patients receiving hemodialysis and potentially eligible for a living donor transplant found that women were less interested than men^6^. Therefore, it could be asked whether this refusal might explain the gender/sex disparities found in previous studies showing lower access to the KT waiting list for > 60-year-old women^7,8^.

Several factors have emerged to explain the patient’s reluctance to undergo KT. A study on Iranian patients receiving peritoneal dialysis reported that the negative consequences of KT and the waiting time, which is often too long, are some of the reasons for refusing this procedure^9^. A study in Brunei Darussalam showed that patients on dialysis refuse KT mainly because they feel well on dialysis and do not want to take risks^10^. For some patients, religious considerations may justify their attitude to KT^11^. In a study on African Americans, women were more positive than men about the experience of dialysis and were often reluctant to undergo KT^12^. Another study on African American urban patients on hemodialysis found that women were less likely to want a living donor kidney transplantation compared with men^6^. A qualitative study in France found that some patients do not trust KT and refuse it. Moreover, the perception of KT were slightly different between men and women^3^.

Nevertheless, the question of the refusal to be registered on the KT waiting list has not been thoroughly studied in France. Previous studies in France on this issue used either a qualitative^13^ or a quantitative method^4^; however, a mixed methods approach should be more suitable^14,15^. Similarly, the determinants and reasons for this refusal are not very well known in France where there is universal health coverage and KT costs are fully covered, as well as the role of gender/sex.

Our aim was to study the refusal of registration on the KT waiting list by patients on dialysis in France using a mixed methods approach. The objective of the quantitative analysis was to describe patient features associated with this refusal. The objective of the qualitative analysis was to identify the reasons for this refusal from the patients and nephrologists’ perspectives. This study is part of a larger project on women’s access to KT in France^3,7,16^.

## Results

### Quantitative results

**Fig. 1:**
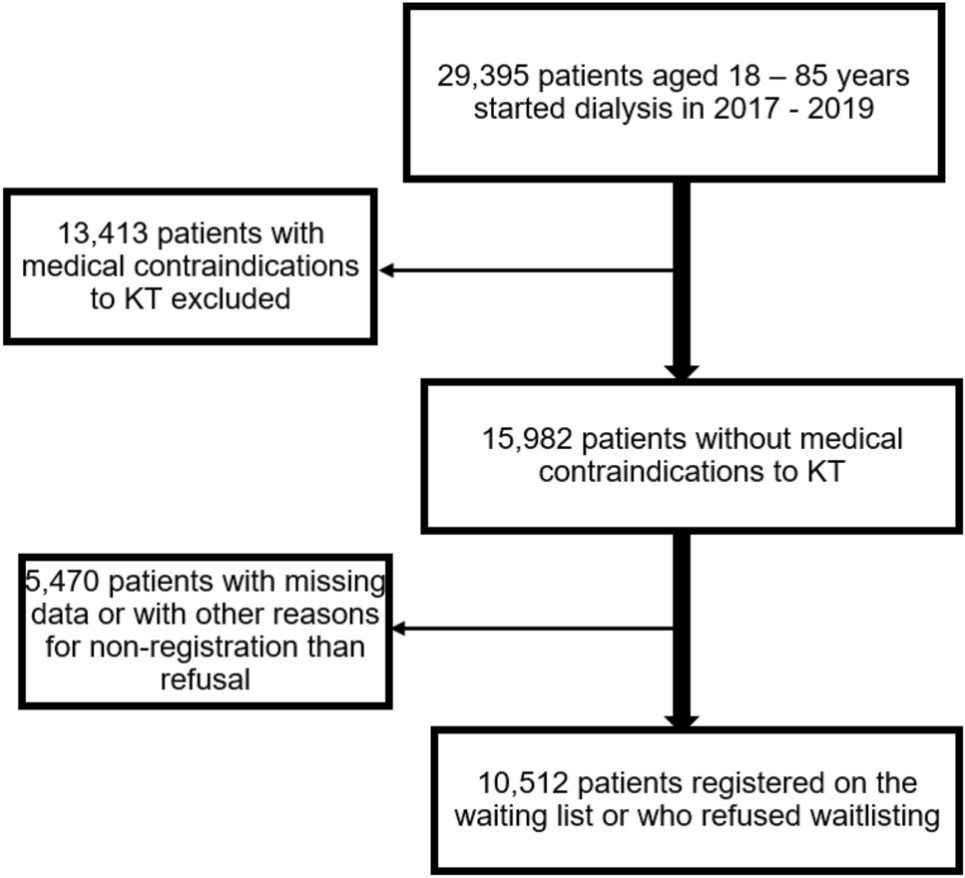
Patients’ selection flowchart.

The mean age of the 10,512 patients included (8.18% of whom had refused to be registered in the KT waiting list) was 57.5 ± 14 years (70.3 ± 9 years for those who refused and 56.4 ± 14 years for those registered in the waiting list) (shown in Fig. 1). Women represented 35.2% (43.6% of the patients who refused registration in the waiting list and 34.4% of those registered). The first dialysis session was carried out autonomously by 7.1% of patients (9.7% of those who refused and 6.9% of those registered). Moreover, 86.1% of patients (89.4% of those who refused and 85.8% of those registered) received hemodialysis; 90.0% of all patients (86.6% of patients who refused and 90.4% of registered patients) walked autonomously, 6.1% (12.9% and 5.5%) had at least three cardiovascular diseases, 3.5% (2.1% and 3.6%) had a liver disease, 35.4% (51.0% and 34.0%) had diabetes, and 1.4% (2.7% and 1.2%) had a psychiatric disorder (Table 1).

**Table 1:** Description of the patients’ characteristics according to their status (registered or refused registration in the kidney transplant waiting list).

	Registered (N = 9652)	Refused (N = 860)	Total (N = 10512)	p-value
**Gender/sex**				< 0.001
Men	6327 (65.6%)	485 (56.4%)	6812 (64.8%)	
Women	3325 (34.4%)	375 (43.6%)	3700 (35.2%)	
**Age group (years)**				< 0.001
18–39	1408 (14.6%)	4 (0.5%)	1412 (13.4%)	
40–59	3758 (38.9%)	107 (12.4%)	3865 (36.8%)	
60–69	2872 (29.8%)	246 (28.6%)	3118 (29.7%)	
70–79	1552 (16.1%)	393 (45.7%)	1945 (18.5%)	
80–85	62 (0.6%)	110 (12.8%)	172 (1.6%)	
**Age (years)**				< 0.001
Mean (SD)	56.36 (13.82)	70.28 (9.38)	57.50 (14.04)	
Median (Q1, Q3)	58.5 (47.6, 67.2)	71.8 (65.1, 77.3)	59.9 (48.5, 68.4)	
**Activity status**				< 0.001
Active	2322 (24.1%)	47 (5.5%)	2369 (22.5%)	
Inactive	5557 (57.6%)	750 (87.2%)	6307 (60.0%)	
Missing	1773 (18.4%)	63 (7.3%)	1836 (17.5%)	
**EDI**				0.02
Other	5632 (58.4%)	553 (64.3%)	6185 (58.8%)	
Most deprived	3601 (37.3%)	297 (34.5%)	3898 (37.1%)	
Missing	419 (4.3%)	10 (1.2%)	429 (4.1%)	
**Hemoglobin (g/dl)**				0.3
< 10	4259 (44.1%)	396 (46.0%)	4655 (44.3%)	
[10_12]	4027 (41.7%)	364 (42.3%)	4391 (41.8%)	
> 12	1002 (10.4%)	77 (9.0%)	1079 (10.3%)	
Missing	364 (3.8%)	23 (2.7%)	387 (3.7%)	
**Albumin (g/dl)**				0.2
< 30	1490 (15.4%)	150 (17.4%)	1640 (15.6%)	
≥ 30	7023 (72.8%)	632 (73.5%)	7655 (72.8%)	
Missing	1139 (11.8%)	78 (9.1%)	1217 (11.6%)	
**BMI (kg/m**^**2**^**)**				0.09
< 18.5	335 (3.5%)	25 (2.9%)	360 (3.4%)	
[18.5;23[	1940 (20.1%)	187 (21.7%)	2127 (20.2%)	
[23;25[	1295 (13.4%)	115 (13.4%)	1410 (13.4%)	
[25;30]	2737 (28.4%)	247 (28.7%)	2984 (28.4%)	
≥ 30	1997 (20.7%)	224 (26.0%)	2221 (21.1%)	
Missing	1348 (14.0%)	62 (7.2%)	1410 (13.4%)	
**Walking impairment**				< 0.001
Cannot walk	55 (0.6%)	14 (1.6%)	69 (0.7%)	
Walk with assistance	203 (2.1%)	43 (5.0%)	246 (2.3%)	
Autonomous	8721 (90.4%)	745 (86.6%)	9466 (90.0%)	
Missing	673 (7.0%)	58 (6.7%)	731 (7.0%)	
**Number of cardiovascular diseases**				< 0.001
0	6675 (69.2%)	394 (45.8%)	7069 (67.2%)	
1	1701 (17.6%)	208 (24.2%)	1909 (18.2%)	
2	743 (7.7%)	147 (17.1%)	890 (8.5%)	
≥ 3	533 (5.5%)	111 (12.9%)	644 (6.1%)	
**Respiratory insufficiency**				< 0.001
No	8526 (88.3%)	720 (83.7%)	9246 (88.0%)	
Yes	890 (9.2%)	111 (12.9%)	1001 (9.5%)	
Missing	236 (2.4%)	29 (3.4%)	265 (2.5%)	
**Active malignancy**				0.9
No	9119 (94.5%)	813 (94.5%)	9932 (94.5%)	
Yes	343 (3.6%)	30 (3.5%)	373 (3.5%)	
Missing	190 (2.0%)	17 (2.0%)	207 (2.0%)	
**Liver disease**				0.02
No	9001 (93.3%)	804 (93.5%)	9805 (93.3%)	
Yes	351 (3.6%)	18 (2.1%)	369 (3.5%)	
Missing	300 (3.1%)	38 (4.4%)	338 (3.2%)	
**Diabetes**				< 0.001
No	6322 (65.5%)	416 (48.4%)	6738 (64.1%)	
Yes	3279 (34.0%)	439 (51.0%)	3718 (35.4%)	
Missing	51 (0.5%)	5 (0.6%)	56 (0.5%)	
**Psychiatric disorder**				< 0.001
No	8981 (93.0%)	778 (90.5%)	9759 (92.8%)	
Yes	119 (1.2%)	23 (2.7%)	142 (1.4%)	
Missing	552 (5.7%)	59 (6.9%)	611 (5.8%)	
**Number of physical disabilities**				< 0.001
0	9275 (96.1%)	801 (93.1%)	10,076 (95.9%)	
≥ 1	377 (3.9%)	59 (6.9%)	436 (4.1%)	
**Primary kidney disease**				< 0.001
Diabetes	1890 (19.6%)	228 (26.5%)	2118 (20.1%)	
Glomerulonephritis	1830 (19.0%)	84 (9.8%)	1914 (18.2%)	
Hypertensive and vascular disease	1745 (18.1%)	208 (24.2%)	1953 (18.6%)	
Other/Unknown	2604 (27.0%)	251 (29.2%)	2855 (27.2%)	
Pyelonephritis	339 (3.5%)	43 (5.0%)	382 (3.6%)	
Polycystic disease	1244 (12.9%)	46 (5.3%)	1290 (12.3%)	
**Ownership of nephrology facility**				< 0.001
Missing	7 (0.1%)	1 (0.1%)	8 (0.1%)	
Private for profit	2664 (27.6%)	221 (25.7%)	2885 (27.4%)	
Private not for profit	1843 (19.1%)	191 (22.2%)	2034 (19.3%)	
Public non-academic	2541 (26.3%)	280 (32.6%)	2821 (26.8%)	
Public academic	2597 (26.9%)	167 (19.4%)	2764 (26.3%)	
**Facility performing kidney transplantation**				0.005
No	7878 (81.6%)	735 (85.5%)	8613 (81.9%)	
Yes	1774 (18.4%)	125 (14.5%)	1899 (18.1%)	
**First dialysis session autonomous**				0.003
No	8943 (92.7%)	775 (90.1%)	9718 (92.4%)	
Yes	663 (6.9%)	83 (9.7%)	746 (7.1%)	
Missing	46 (0.5%)	2 (0.2%)	48 (0.5%)	
**Emergency dialysis start**				0.3
No	7380 (76.5%)	653 (75.9%)	8033 (76.4%)	
Yes	1881 (19.5%)	180 (20.9%)	2061 (19.6%)	
Missing	391 (4.1%)	27 (3.1%)	418 (4.0%)	
**First dialysis with catheter**				0.7
No	4929 (51.1%)	446 (51.9%)	5375 (51.1%)	
Yes	3778 (39.1%)	351 (40.8%)	4129 (39.3%)	
Missing	945 (9.8%)	63 (7.3%)	1008 (9.6%)	
**Dialysis modality**				0.003
Hemodialysis	8281 (85.8%)	769 (89.4%)	9050 (86.1%)	
Peritoneal dialysis	1371 (14.2%)	91 (10.6%)	1462 (13.9%)	
**French region**				< 0.001
Auvergne Rhône Alpes	1109 (11.5%)	76 (8.8%)	1185 (11.3%)	
Bourgogne Franche Comté	364 (3.8%)	16 (1.9%)	380 (3.6%)	
Bretagne	404 (4.2%)	28 (3.3%)	432 (4.1%)	
Centre Val de Loire	402 (4.2%)	18 (2.1%)	420 (4.0%)	
Corse	34 (0.4%)	3 (0.3%)	37 (0.4%)	
Grand Est	723 (7.5%)	128 (14.9%)	851 (8.1%)	
Hauts de France	631 (6.5%)	128 (14.9%)	759 (7.2%)	
Île-de-France	2490 (25.8%)	38 (4.4%)	2528 (24.0%)	
Normandie	457 (4.7%)	65 (7.6%)	522 (5.0%)	
Nouvelle Aquitaine	819 (8.5%)	65 (7.6%)	884 (8.4%)	
Occitanie	860 (8.9%)	88 (10.2%)	948 (9.0%)	
Pays de la Loire	467 (4.8%)	59 (6.9%)	526 (5.0%)	
Provence Alpes Côte Azur	621 (6.4%)	90 (10.5%)	711 (6.8%)	
Guadeloupe	50 (0.5%)	4 (0.5%)	54 (0.5%)	
Martinique	61 (0.6%)	4 (0.5%)	65 (0.6%)	
French Guiana	27 (0.3%)	0 (0.0%)	27 (0.3%)	
Reunion	128 (1.3%)	49 (5.7%)	177 (1.7%)	
Mayotte	5 (0.1%)	1 (0.1%)	6 (0.1%)	

In the multivariate regression analysis, being a woman (adjOR 1.83 [1.56 – 2.14]), being older than 60 years (adjOR 5.64 [4.31—7.39]), having started dialysis autonomously (adjOR 1.59 [1.21—2.09]), diabetes (adjOR 1.35 [1.11—1.64]), psychiatric disorders (adjOR 2.04 [1.23—3. 38]), and cardiovascular diseases (adjOR for 1 disease: 1.53 [1.26—1.84], adjOR for 2 diseases: 2.25 [1.80—2.81], adjOR for ≥ 3 diseases: 2.17 [1.68—2.79]) increased the odds of refusing registration in the waiting list. Compared with patients with polycystic kidney disease, those with diabetic nephropathy (adjOR 1.62 [1.11 – 2.36]), hypertensive or vascular nephropathy (adjOR 2.01[1.42—2.84]), pyelonephritis (adjOR 3.17 [2.0—5.0]), or unknown or other initial nephropathy (adjOR 1.94 [1.38—2.73]) were more likely to refuse waitlisting.

Being on peritoneal dialysis (adjOR 0.77 [0.60 – 0.99]), having a liver disease (adjOR 0.57 [0.34—0.95]) and walking autonomously (adjOR 0.44 [0.23—0.87]) reduced the odds of refusing registration. Compared with patients who started dialysis in a public non-university center, patients who started dialysis in a private for-profit center (adjOR 0.72 [0.59—0.87]) or in a public university center (adjOR 0.58 [0.42—0.79]) were less likely to refuse registration in the waiting list (Table 2).

**Table 2:** Multivariate logistic model to identify factors associated with registration refusal by patients on dialysis.

	**Odds ratio**	**P-value**	**[95% -**	**CI]**
Gender/sex				
Men	1			
Women	1.83	< 0.01	1.56	2.14
Age group (years)				
< 60	1			
≥ 60	5.64	< 0.01	4.31	7.39
EDI				
Other	1			
Most deprived	0.93	0.38	0.80	1.09
Activity status				
Active	1			
Inactive	1.51	0.09	0.92	2.46
Hemoglobin (g/dl)				
< 10	1			
[10_12]	0.92	0.32	0.78	1.08
> 12	0.92	0.54	0.70	1.21
Albumin (g/dl)				
< 30	1			
≥ 30	0.97	0.77	0.79	1.19
BMI (kg/m^**2**^**)**				
< 18.5	1			
[18.5;23[	1.30	0.25	0.83	2.03
[23;25[	1.07	0.78	0.66	1.72
[25;30]	0.90	0.64	0.56	1.42
≥ 30	0.98	0.95	0.62	1.55
Number of physical disabilities				
0	1			
≥ 1	1.33	0.09	0.95	1.85
Liver disease				
No	1			
Yes	0.57	0.03	0.34	0.95
First dialysis session autonomous				
No	1			
Yes	1.59	< 0.01	1.21	2.09
Number of cardiovascular diseases				
0	1			
1	1.53	< 0.01	1.26	1.84
2	2.25	< 0.01	1.80	2.81
≥ 3	2.17	< 0.01	1.68	2.79
Primary kidney disease				
Polycystic disease	1			
Diabetes	1.62	0.01	1.11	2.36
Glomerulonephritis	1.35	0.12	0.92	1.98
Hypertensive and vascular disease	2.01	< 0.01	1.42	2.84
Other/Unknown	1.94	< 0.01	1.38	2.73
Pyelonephritis	3.17	< 0.01	2.01	5.00
Walking impairment				
Cannot walk	1			
Walk with assistance	0.65	0.27	0.31	1.39
Autonomous	0.44	0.02	0.23	0.87
Respiratory insufficiency				
No				
Yes	1.00	0.98	0.79	1.26
Active malignancy				
No	1			
Yes	0.72	0.099	0.49	1.06
Diabetes				
No	1			
Yes	1.35	0.003	1.11	1.64
Psychiatric disorder				
No	1			
Yes	2.04	0.006	1.23	3.38
Ownership of nephrology facility				
Public not university center	1			
Private for profit	0.72	0.001	0.59	0.87
Private not for profit	0.90	0.321	0.72	1.11
Public university center	0.58	0.001	0.42	0.79
Facility performing kidney transplantation				
No	1			
Yes	1.08	0.65	0.77	1.51
Emergency dialysis start				
No	1			
Yes	1.06	0.550	0.87	1.31
First dialysis with catheter				
No	1			
Yes	1.12	0.204	0.94	1.34
Dialysis modality				
Hemodialysis	1			
Peritoneal dialysis	0.77	0.046	0.60	0.99

EDI was not associated with registration refusal (adjOR 0.93 [0.80 – 1.09]).

There was a significant association between age and gender/sex (adjOR 1.84 [1.20 – 2.81]). In the < 60-year-old patient group, there was no gender/sex difference in the registration refusal rate (adjOR 1.16 [0.77 – 1.75]). Only ≥ 60-year-old women were more likely to refuse registration (adjOR: 2.01 [1.70 – 2.39]).

No interaction was found between gender/sex and EDI (adjOR 0.94 [0.69 – 1.29]), liver disease (adjOR 1.13 [0.42 – 3.03]), walking impairment (adjOR 0.70 [0.19 – 2.57]), or autonomous first dialysis session (adjOR 0.88 [0.52 – 1.47]).

### Qualitative results

Interviews, on average, lasted 30 min. Twenty-one patients (13 women and 8 men) and 11 nephrologists (4 women and 7 men) were included. Four themes were identified: “Transplant refusal: a question of age?”, “Place of gender/sex in transplant refusal”, “Context of dialysis start and feelings about dialysis”; and “Role of comorbidities, experiences and fears on the patient’s decision”.

#### Theme 1: Transplant refusal: a question of age?

Several patients felt that younger patients were more deserving than they were because they had already lived a good part of their life. This feeling was strengthened when the question of living donor transplantation was raised. *"I think that maybe there are younger people who deserve a transplant more than I do". ****PT1 (76-year-old woman).*** Other older patients refused registration without any real reason other than their age. For nephrologists, advanced age did not represent a typical profile of patients who refuse transplantation, although it could be a deterrent. A nephrologist said *“But there are still people who are afraid of transplants, especially in the… older people.” ****N11 (nephrologist, woman)*** and another elaborated *"I have a patient on dialysis, a very old patient who has been on dialysis since the 1980s, and a young patient on dialysis who work, and they don’t want transplants”. ****N9 (nephrologist, woman***). Additional illustrative quotes related to this theme are in Table 3.

**Table 3:** Selected illustrative quotes related to the themes.

Themes	Quotes
Theme 1: “**Transplant refusal: a question of age?”**	*“Transplants are not for old people. It’s better to keep them for younger people because at my age it’s no longer… I think that it’s not useful, what’s the point?” ****PT2 (***≥ ***60-year-old man)*** *“Because I’m too old and then if there is a young girl…when I see the…I met a young 23-year-old girl who had kidney problems, I would rather leave it to her than to me; and my children know this.” ****PT3 (***≥ ***60-year-old woman)*** *“So, what stopped me, I said: ‘I’m 76, I am not going to be bothered with this’. For me… no. At the moment, I do not accept the transplant, at the time T.” ****PT4 (***≥ ***60-year-old man)*** *“At my age, no. And then with all what must be done…no, no”. ****PT5 (***≥ ***60-year-old woman)*** *“Indeed, some patients will say to me ‘I am 65, it is better if I leave the graft for somebody […] who is younger”. ****N8 (nephrologist, man)***
Theme 3: “**Context of dialysis start and feelings about dialysis”**	*“But let’s say that as the dialysis is going very well and I can still come regularly without any problem in the morning, every other day, so there is no real problem for me. However, if I were 20 years younger, maybe I might react differently. But now, well, it’s not that I’m defeatist, but given that we have here, luckily, a service that is very supportive and reassuring and that we are very well looked after, and that there has never been a problem, well, the catheter works very well, I keep my finger crossed (laughs) for the moment, so…well, we are very well looked after, so that must be what makes me hesitant about the transplant.” ****PT1 (*≥ *60-year-old woman)*** *“In fact, at the beginning they are interested, then, in fact, they start dialysis, they find their balance, in between brackets, well… and that suits them, in between brackets, even if it is constraining. They get used, it reassures them. And in fact, afterwards, they say to me: ‘Well, no, I’m fine like this’.” ****N1 (nephrologist, woman)*** *“Oh yes, that’s it. I’m happy with it because I organize myself, I have my little snack, I have my computer. It also allows me… I also see it like that, that it allows me to have some time for myself when I’m in quietness, I’m in my own bubble, and it is my own time, no one to bother me.” ****PT6 (*< *60- year-old man)*** *“I tried to go through the whole thing to understand what his reasons were. He said that he felt well on dialysis. This a patient on nocturnal dialysis, three times per week, who work 100% and has a fulfilled family life and who did not want to experience any instability due to the transplant.” ****N3 (nephrologist, man)***
Theme 4: “**Role of comorbidities, experiences and fears on the patient’s decision”**	*“Yes, yes because I don’t want that, that’s it. When you are blocked for ten days and they tell you: ‘You must not move from your bed’. I could not even seat on the edge of the bed, no, no. What’s more, during the COVID, so I could not see a soul, I was all alone in the room. No, no, I’m over with that…no, no, that’s it.” ****PT9 (*< *60 year-old woman)*** *“Right so, with the doctors’ mistrust already. Because I feel that I have been already betrayed for years, I find that they are not trustworthy. And then… no, I am not ready.” ****PT10 (*< *60 year-old woman)*** *“In fact, I think that I went through so many operations that now I tell myself: can my body accept such a big surgical intervention.” ****PT7 (*< *60 year-old woman)*** *“Another… another type of refusal, it is for example somebody who had already a transplant twice, it did not go well, this patient does not want another transplant, he’s fine on dialysis, those are the reasons.” ****N10 (nephrologist, man)*** *“Well, I gave you my opinion, I don’t see myself going through such an operation. It’s very very…very hard, I think, to… the kidney transplant.” ****PT1 (*≥ *60 year-old woman)*** *“I can’t stand drugs: all drugs have negative effects. So, when somebody comes to see us and lists the drugs that we must take for a transplant all our life, at a precise time and the negative effects, I know that the negative effects, it will be not for the positive side of the transplant, it will be the negative side that I can’t stand.” ****PT10 (*< *60 year-old woman)***

#### Theme 2: Place of gender/sex in transplant refusal

A woman said *“… in fact, I have many nightmares. I wake up a lot at night, I have nightmares, I see the operation going badly, I see myself leaving my daughter, I don’t know what to do”* **PT7 (< 60-year-old woman)**. The emotional bond between this woman and her child prevented her from accepting the transplant. However, the role of gender/sex was not clearly identified in the interviews. Refusal was expressed by men and women. One nephrologist stated, *"But on the question of transplant refusal, I don’t get the impression that we have more women refusing to initiate the assessment or to be registered."* **N4 (nephrologist, woman)**. Yet, a nephrologist thought that women refused more to be registered than men: *"Strangely, I have the impression that it’s more women who refuse transplants"* **N5 (nephrologist, man)**. This perception was not shared by another nephrologist: *"No, the examples I can remember… the last examples I can remember, recent or old, but the ones that struck me were men. So it wasn’t women, because sometimes people say the opposite, and in fact for me it was men."* **N6 (nephrologist, man).**

#### Theme 3: Context of *dialysis* start and feelings about *dialysis*

In the interviews, several patients mentioned the fact that they felt better on dialysis as a reason for not taking the risk of KT. A nephrologist said: *“We have patients who have been on dialysis for a very long time and who are very happy on dialysis, who maintain social contacts with the dialysis team, and so they absolutely don’t want to lose this social link.” ****N1 (nephrologist, man)****.* This feeling was often reinforced by uncertainty about the KT outcome. Patients felt they had found a balance that they were not prepared to give up, especially because the alternative involved a great deal of uncertainty. One patient mentioned the role of his professional activity (co-owner of a company) in his decision to refuse KT. *"My doctors keep bugging me about it. But at the moment I can’t because I’ve got too much work and I can’t afford to be off work for a while like that. Today, I'm carrying the company at arm’s length and if I’m not here tomorrow, well, unfortunately it could be complicated.” ****PT6 (*< *60-year-old man).*** Additional illustrative quotes related to this theme are in Table 3**.**

#### Theme 4: Role of comorbidities, experiences and fears on the patient’s decision

Past experiences concerning other comorbidities contributed to the patients’ decision to refuse registration. Patients felt that they had suffered enough and were not ready to embark on a new difficult journey. Patients also questioned their body ability to withstand all that entails a KT. They saw their bodies as fragile, due to comorbidities, and unable to endure the transplant procedure. Some patients justified their refusal due to KT failure in relatives. Faced with the uncertainty about the KT outcome, some patients sought external feedback to form their opinion.* "All the people I know who had this problem, all had problems with the graft, and not one graft has lasted.****" PT38 (*< *60 year-old woman).*** Other patients refused KT because they had a personal history of a transplant that did not go well. The fear of KT surgery complications and the side effects of the drugs that must be taken after the operation were cited by several patients as reasons for their refusal. This feeling was reinforced when it was shared also by relatives and children. Additional illustrative quotes related to this theme are in Table 3**.**

### Integration of the results

Table 4 presents the results from the integration of the findings from the two analyses, highlighting the areas of convergence, divergence and expansion. The two analyses converged at many points showing that older patients, women, patients who were autonomous and patients who had previous difficult experiences, mainly linked to comorbidities, were more likely to refuse KT. However, there were also areas of divergence. A male nephrologist thought that men refused more frequently registration than women, unlike the quantitative analysis results. Similarly, a divergence concerned autonomy. Patients on peritoneal dialysis tended to refuse registration less frequently than patients on other dialysis modalities. Lastly, an area of expansion concerned the finding that liver disease as co-morbidity decreased the odds of refusing registration, unlike other comorbidities.

**Table 4:** Integration of the findings of the quantitative and qualitative analyses.

Major topics	Quantitative results	Qualitative results	Mixed methods comparison
Age	AdjOR of ≥ 60-year-old compared with < 60-year-old patients: 5.64 [4.31—7.39]	**Theme 1: Transplant refusal: a question of age?** *"I think that maybe there are younger people who deserve a transplant more* *than I do". ****PT1 (76-year-old woman)*** *“Transplants are not for old people. It’s better to keep them for younger people* *because at my age it’s no longer… I think that it’s not useful, what’s the point?” ****PT2*** ***(***≥ ***60-year-old man)*** *“But there are still people who are afraid of transplants,* *especially in the… older people.” ****N11 (nephrologist, woman)***	The two results **converge** Older patients refuse transplants more than younger patients
Gender/Sex	AdjOR of women compared with men: 1.83 [1.56 – 2.14]) AdjOR of > 60-year-old women compared with > 60-year-old men: 2.01 [1.70 – 2.39]	**Theme 2: Place of gender/sex in transplant refusal** *“… in fact, I have many nightmares. I wake up a lot at night, I have nightmares, I see the operation going badly, I see myself leaving my daughter, I don’t know what to do”* **PT7 (< 60-year-old woman)** *"But on the question of transplant refusal, I don’t get the impression that we have more women refusing to initiate the assessment or to be registered."* **N4 (nephrologist, woman)** *"No, the examples I can remember… the last examples I can remember, recent or old, but the ones that struck me were men. So it wasn’t women, because sometimes people say the* *opposite, and in fact for me it was men."* **N6 (nephrologist, man)**	There is **convergence** between the qualitative and quantitative findings showing that women refuse registration much more frequently However, there is also **divergence** because a male nephrologist thought that men refused KT more often
Context of dialysis	AdjOR of patients who started dialysis autonomously compared with those who did not: 1.59 [1.21—2.09] AdjOR of patients on peritoneal dialysis compared with those on hemodialysis: 0.77[0.60 – 0.99]	**Theme 3: Context of dialysis start and feelings about dialysis** *“In fact, at the beginning they are interested, then, in fact, they start dialysis, they find their balance, in between brackets, well… and that suits them, in between brackets, even if it is constraining. They get used, it reassures them. And in fact, afterwards, they say to me: ‘Well, no, I’m fine like this’.” ****N1 (nephrologist, woman)*** *“Oh yes, that’s it. I’m happy with it because I organize myself, I have my little snack, I have my computer. It also allows me… I also see it like that, that it allows me to have some time for myself when I’m in quietness, I’m in my own bubble, and it is my own time, no one to bother me.” ****PT6 (*< *60- year-old man)***	There is **convergence** between the results showing that patients who are autonomous concerning dialysis refuse registration much more often However, there is **divergence** between the analyses concerning peritoneal dialysis
Comorbidities and past experiences	**Diabetes** adjOR:1.35 [1.11—1.64] **Psychiatric disorders** adjOR: 2.04 [1.23—3. 38] **Cardiovascular diseases** 1 disease:1.53 [1.26—1.84] 2 diseases: 2.25 [1.80—2.81], ≥ 3 diseases: 2.17 [1.68—2.79]) increased the odds of refusing registration on the waiting list **Liver disease** adjOR: 0.57 [0.34—0.95]	**Theme 4: Role of comorbidities, experiences and fears on the patient’s decision** *“In fact, I think that I went through so many operations that now I tell myself: can my body accept such a big surgical intervention.” ****PT7 (*< *60 year-old woman)*** *"I know someone who had three transplants and it didn’t* *work. So, considering what I’ve got, well, no****" PT8 (***≥ ***60 year-old man)***	There are areas of **convergence** between the results showing that patients who had previous difficult experiences, probably linked to co-morbidities, are much more likely to refuse registration There is also an area of **expansion**: patients liver disease as co-morbidity are more likely to accept registration. This finding deserves to be better explored

## Discussion

This study used a convergent mixed methodological approach^17^ that combined a quantitative and a qualitative analysis to better understand the reasons of refusing registration on the KT waiting list in France. To our knowledge, this is the first study that used this methodology to investigate the issue of registration refusal. Unlike what one might expect, not all patients eligible for KT want to begin the transplant process and therefore, refuse to be placed on the waiting list. This study also identified some factors associated with this refusal. Most of these factors converged across the two (quantitative and qualitative) analyses. These included age, gender/sex, autonomy on dialysis and comorbidities. The integration of results also found some divergence concerning gender/sex and autonomy and highlighted an area of expansion about comorbidities.

The role of age in their decision was mentioned by several patients. Some patients felt that they were unable to cope with a KT because of their advanced age. Moreover, some older patients felt that younger patients needed a transplant more than they did. The quantitative analysis supported these results by showing that ≥ 60-year-old patients were more likely to refuse a transplant than younger patients (adjOR: 6.35 [4.73—8.53]), even in the absence of formal medical contraindications. Indeed, with age, comorbidities also increase and patients are less willing to take risks. This is in line with a qualitative study carried out in France^13^. A qualitative study in the United Kingdom reported that most interviewed patients thought that younger patients needed to be prioritized for KT^30^. This could be partly explained by the risk aversion of older patients and their altruism towards younger people. This result converged in the two analyses, although some nephrologists were skeptical about the role of age.

Our study showed that feeling well on dialysis was a reason for refusing registration on the KT waiting list. This was also noted in a study that summarized the qualitative evidence on this topic^31^. Specifically, patients who started dialysis autonomously were more likely to refuse to be placed on the KT waiting list. A qualitative study of patients undergoing nocturnal home dialysis in Canada also found that autonomy during dialysis was a determining factor in the patients’ choice^32^. A qualitative study in Australia on the opinion of patients with stage 5 CKD on the different replacement treatments reported that patients preferred the treatment giving them the most autonomy. Thus, if the dialysis modality is not perceived as restrictive, motivation for KT is not strong^33^. However, unlike what found in the literature^5^, in the quantitative part of our study, patients on peritoneal dialysis were 23% less likely to refuse registration on the waiting list, which diverged from the qualitative study findings. A qualitative study, focusing on patients receiving peritoneal dialysis will help to address this discrepancy.

Our study highlighted the role of gender/sex in the patients’ choice. Our quantitative analysis showed that women were more likely to refuse transplant than men. Similarly, among urban African American patients on hemodialysis, women were less likely to accept a living donor kidney transplant^6^. The factors underlying these results were not clearly identified in the interviews. A woman justified her refusal by the fear of leaving her child alone if the operation went wrong. Similarly, a study in Canada found that women could refuse a transplant just to be able to look after their child, especially if the child was young or if she was the *“primary income earner in her family”*^32^. Theories explaining women refusal highlighted economic reasons^34,35^ and giving priority to men^36^. However, as in France, the KT cost is fully covered by the social security system, the economic reason should not apply. This was confirmed by our quantitative analysis that did not find any association between EDI and refusal or between EDI and gender/sex. While, most of the qualitative data converged with the quantitative results, a nephrologist thought that the patients’ gender/sex did not influence their choice. Another male nephrologist thought that men were more likely to refuse registration. The nephrologists’ gender/sex was not taken into account in the analysis of the qualitative data, but it could shed some light on this divergence. These results highlighted the need for standardized guidelines in discussing KT with patients, taking gender perspectives into account.

The qualitative analysis demonstrated the role of comorbidities. Patients who had a previous medical or surgical experience that had a major impact on their health were more likely to refuse KT. In agreement, the quantitative analysis found a link between diabetes and number of cardiovascular diseases and KT refusal, even in the absence of formal medical contraindications. This result cannot be generalized to all comorbidities. Indeed, the quantitative analysis found that patients with liver disease or who walked autonomously were less likely to refuse registration on the KT waiting list. As liver disease is not a very disabling disease at the beginning, this result might be explained by the fact that these patients did not consider their condition to be deteriorated and therefore, did not refuse the KT process, unlike patients with cardiovascular diseases (stroke, myocardial infarction, peripheral artery occlusive disease) who require extensive care and have many constraints. Similarly, the qualitative analysis showed that patients who perceived their state of health as fragile or who had a long medical history were more likely to refuse to be placed on the waiting list despite the nephrologist’s offer. The literature on the link between comorbidities and transplant refusal is very limited. Some patients reported fear or uncertainty of the transplant procedure as a reason for refusing KT, which may indicate insufficient or unclear patient information.

This study adopted a convergent mixed methods approach that combined a quantitative and a qualitative analysis^17^. This allowed thoroughly exploring the topic by drawing on the strengths of each approach^15^. Although original, this study has some limitations. Some of the results diverged depending on the approach considered and deserve to be better explored. Recording of the waiting list registration refusal variable in the REIN database could be biased. This variable is recorded by the nephrologist each year and the patient’s decision could have changed over time. Also, the existence of comorbidities may lead a nephrologist to insist more on the KT risks and therefore, influence the patient’s decision. Moreover, patients who were undergoing the transplant assessment were excluded from the quantitative analysis. This was justified by the fact that the decision had not been made yet and taking them into account could have led to an underestimation of the refusal rate. However, this could have caused a selection bias. In this study, we were mainly interested in the role of individual characteristics in refusal. However, regional characteristics such as healthcare practices, resource availability and patient education across regions could impact decisions, and analyzing regional variations in refusal rates might reveal important location-specific influences. Finally, the use of a gender analysis framework could provide a better understanding of the differences between men and women concerning KT refusal. Given the robustness of our methodology and the similarity between the care pathways for transplants of other organs, our results could be generalized beyond KT.

This study found that some patients refused to be registered in the KT waiting list and that their age, gender/sex and comorbidities played an important role in this choice. Most results were supported by both approaches (quantitative and qualitative analyses). Interventions targeting the factors identified in this study will help to improve KT accessibility and reduce disparities. For example, setting up therapeutic education sessions or consultations with a psychologist might enable older women to perceive KT in a different way and accept it.

## Methods

### Study design

This convergent mixed methods study combined a quantitative analysis and a qualitative analysis^17^ to investigate the same question: the reasons of refusing registration on the KT waiting list. This design allows comparing the results of the two approaches to better explore the study question^18^. The Good Reporting of A Mixed Methods Study (GRAMMS) Checklist was used to ensure accurate reporting^19^.

### Quantitative analysis

#### Study population

A descriptive and analytical cross-sectional analysis was performed using data from patients present in the Renal Epidemiology and Information Network (REIN) registry on 31 December 2022. This registry collects information of all patients on kidney replacement therapy in France^20^. All 2017–2019 dialysis incident patients in France, aged from 18 to 85 years, eligible for KT and who were registered or refused to be registered on the waiting list were included. Patients with other reasons for not being registered on the waiting list were not included (shown in Fig. 1).

### Data collection

The patients’ sociodemographic characteristics, comorbidities and information on their treatment, such as refusal of registration on the KT waiting list, were extracted from the REIN registry that collects data on all patients with chronic kidney disease (CKD) who start a renal replacement therapy in France^20,21^. To capture the patient socio-economics status, the European Deprivation Index (EDI; a neighborhood social deprivation index)^22^ was calculated and added to the REIN registry.

### Statistical analyses

Quantitative variables were presented as mean values with their standard deviations, and categorical variables as numbers and percentages. The Student’s *t*-test and the Chi-2 or Fisher’s exact tests were used to compare quantitative and categorical variables, respectively, in patients who refused to be registered on the waiting list and patients who were registered.

Then, a binary logistic regression method was used with refusal of registration on the KT waiting list as the outcome of interest. This variable is collected at dialysis start and updated every year. For this study, the patient status at year 3 after dialysis start was considered as a binary variable (0 = registered and 1 = refused to be registered). Year 3 after dialysis start was chosen because in France, the KT waiting list registration plateau generally occurs three years after dialysis initiation ^23^. All variables with p-value < 0.20 in univariate analysis were included in the final model. The explanatory variables were age, gender/sex, EDI, activity status, hemoglobin level, albumin level, body mass index (BMI), comorbidities, renal replacement therapy center ownership, emergency dialysis start, walking impairment, first dialysis session characteristics, number of physical disabilities, and KT activity of the dialysis center. Interactions between gender/sex and comorbidities, age and social deprivation were tested. The results of the binary logistic regression analysis were presented as adjusted odd ratios (adjOR) with their 95% confidence interval. Missing data were considered as missed at random and were imputed using Multiple Imputation by Chained Equations with five cycles ^24,25^. The STATA 17 (StataCorp) software was used for the statistical analysis. Additional analyses including all patients undergoing assessment and who had not decided (consent or refusal) about registration in the KT waiting list are in supplementary data (Table S1).

### Qualitative analysis

#### Study population

Twenty-one patients with CKD older than 18 years who started dialysis in 2021 and had refused to be registered in the KT waiting list and 11 nephrologists were purposively selected from three French regions: Bretagne, Normandie and Île-de-France).

### Data collection and analysis

Data were collected through semi-structured interviews that were recorded using a Dictaphone during a dialysis session (patients) or a specific appointment (nephrologists). LKA (MD, PhD with experience in quantitative and qualitative studies) and three researchers (two women and one man) with experience in qualitative studies conducted the interviews. Interviewers were not involved in patient care. The main question to the patients was *“Why did you refuse to be registered on the KT waiting list?”* Interviews with the nephrologists focused on two questions: “*Who are the patients who refuse registration on the KT waiting list?”* and *“Why?”.* Interviews were fully transcribed and a thematic analysis ^26^ was carried out concomitantly by LKA and AC (senior researcher in qualitative studies) to identify the reasons of refusal. The Consolidated Criteria for Reporting Qualitative Research (COREQ) framework was used to ensure the study quality ^27^. Theoretical saturation was reached when the analysis did not bring any additional theme. The Nvivo software (version 1.7.1, QSR international) was used for the initial coding.

### Integration of the quantitative and qualitative analysis findings (Shown in Fig. 2)

**Fig. 2:**
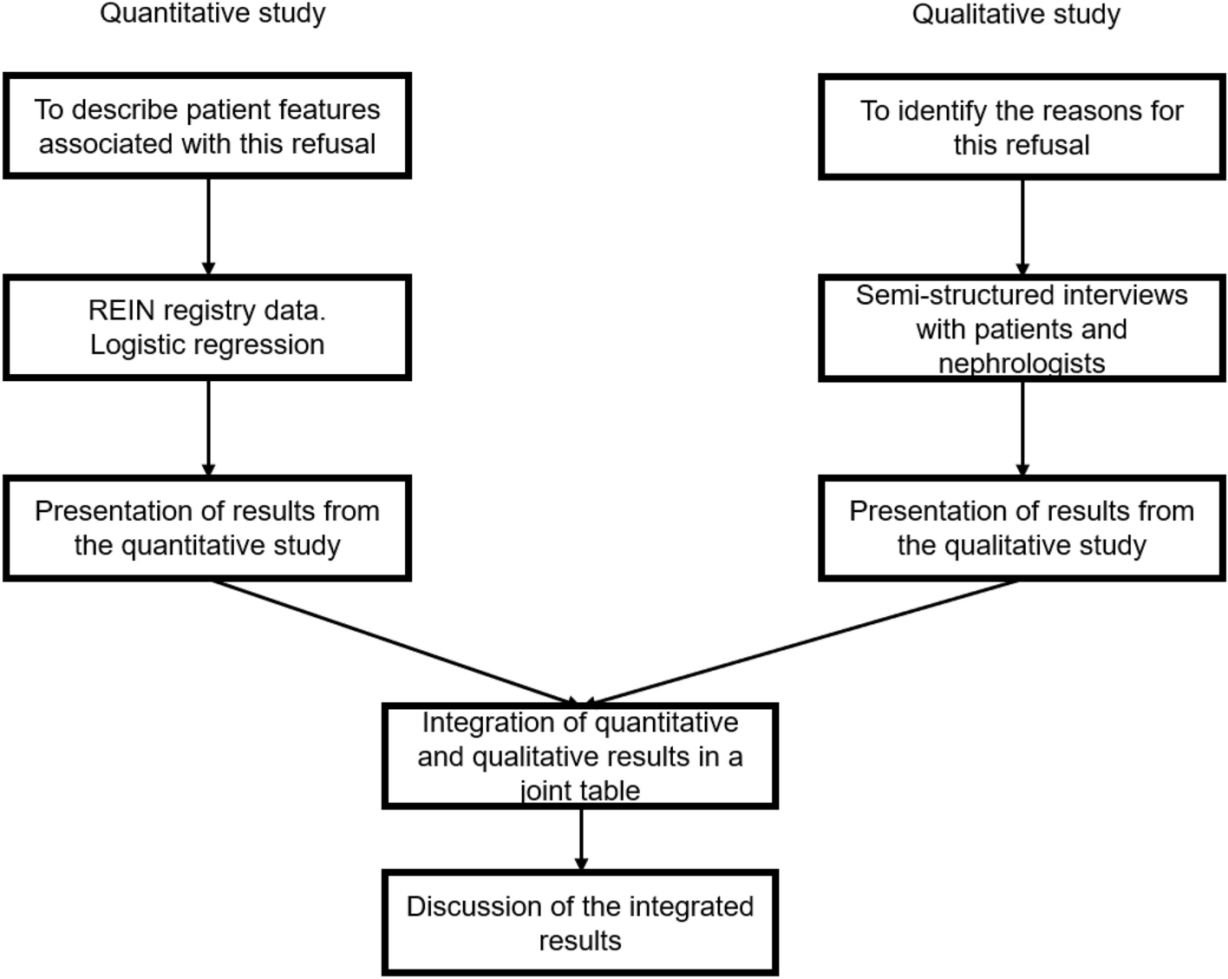
Description of the mixed methods approach used in this study.

The quantitative and qualitative data were analyzed separately and integrated in the form of a joint table in the Results section (shown in Table 4). The integration process involved the comparison of the results of the two analyses to identify convergencies (agreement between the sets of findings), complementarities (different but non-contradictory interpretations), divergencies (conflicting interpretations) and expansions (some findings overlapped but also provided space for further interpretation) ^28,29^.

## Supplementary Information

Supplementary Information.

## Data Availability

The data underlying this article cannot be shared publicly for the privacy of individuals that participated in the study. Please contact Dr Cecile COUCHOUD (cecile.couchoud@biomedecine.fr) for the REIN registry data and Dr ADOLI (Latame.adoli@ehesp.fr) or Dr BAYAT (Sahar.Bayat-Makoei@ehesp.fr) for the qualitative study data.

## References

[1] Kim, D. G. et al. Survival benefit of kidney transplantation in patients with end-stage kidney disease and prior acute myocardial infarction. *Transpl. Int.* **36**, 11491 (2023). 37692454 10.3389/ti.2023.11491 PMC10483068

[2] Wu, H. et al. Economic burden and cost-utility analysis of three renal replacement therapies in ESRD patients from Yunnan Province. *China. Int. Urol. Nephrol.* **52**(3), 573–579 (2020). 32009220 10.1007/s11255-020-02394-1

[3] Adoli, L. K. et al. Experience of chronic kidney disease and perceptions of transplantation by sex. *JAMA Netw. Open* **7**(7), e2424993 (2024). 39083269 10.1001/jamanetworkopen.2024.24993 PMC11292447

[4] Vabret, E. et al. Qui sont ces patients en dialyse non inscrits sur liste d’attente de greffe rénale ?. *Néphrologie Thérapeutique* **16**(3), 139–146 (2020). 32409290 10.1016/j.nephro.2020.02.014

[5] Buturović-Ponikvar, J. et al. Dialysis patients refusing kidney transplantation: data from the Slovenian renal replacement therapy registry. *Ther. Apher. Dial.* **15**(3), 245–249 (2011). 21624070 10.1111/j.1744-9987.2011.00945.x

[6] Gillespie, A. et al. Sex differences and attitudes toward living donor kidney transplantation among urban black patients on hemodialysis. *Clin. J. Am. Soc. Nephrol. CJASN* **9**(10), 1764–1772 (2014). 25125384 10.2215/CJN.12531213 PMC4186525

[7] Adoli, L. K. et al. Lower access to kidney transplantation for women in France is not explained by comorbidities and social deprivation. *Nephrol. Dial Transplant*10.1093/ndt/gfae047ss (2024). 38383847 10.1093/ndt/gfae047 PMC11483620

[8] Couchoud, C., Bayat, S., Villar, E., Jacquelinet, C. & Ecochard, R. Registry on behalf of the R. a new approach for measuring gender disparity in access to renal transplantation waiting lists. *Transplantation.* **94**(5), 513–519 (2012). 22895611 10.1097/TP.0b013e31825d156a

[9] Biabani, F., Rahmani, A., MahmudiRad, G., Hassankhani, H. & Azadi, A. Reasons for kidney transplant refusal among patients receiving peritoneal dialysis: A qualitative study. *Perit. Dial Int.* **43**(5), 395–401 (2023). 36601692 10.1177/08968608221146865

[10] Farah, S. S. et al. Barriers to kidney transplantation as a choice of renal replacement therapy. *Transplant Proc.* **50**(10), 3165–3171 (2018). 30577183 10.1016/j.transproceed.2018.07.005

[11] Cummins, P. J. & Nicoli, F. Justice and respect for autonomy: Jehovah’s witnesses and kidney transplant. *J. Clin. Ethics* **29**(4), 305–312 (2018). 30605440

[12] Salter, M. L. et al. Perceptions about hemodialysis and transplantation among African American adults with end-stage renal disease: inferences from focus groups. *BMC Nephrol.* **16**(1), 49 (2015). 25881073 10.1186/s12882-015-0045-1 PMC4395977

[13] Senghor, A. S. Reasons for dialysis patients choosing or refusing kidney transplantation as renal replacement therapy: A qualitative study. *Néphrologie Thérapeutique* **15**(7), 511–516 (2019). 31668488 10.1016/j.nephro.2019.07.327

[14] Houghton, L. C. & Paniagua-Avila, A. Why and how epidemiologists should use mixed methods. *Epidemiol. Camb. Mass* **34**(2), 175–185 (2023). 10.1097/EDE.0000000000001565 PMC9891266 36722799

[15] Bailey, P. K., Hole, B. D., Plumb, L. A. & Caskey, F. J. Mixed-methods research in nephrology. *Kidney Int.* **101**(5), 895–905 (2022). 35227687 10.1016/j.kint.2022.01.027

[16] Adoli, L. et al. Women’s access to kidney transplantation in france: a mixed methods research protocol. *Int. J. Environ. Res. Public Health* **19**(20), 13524 (2022). 36294104 10.3390/ijerph192013524 PMC9603645

[17] Creswell JW, Plano Clark VL. Designing and conducting mixed methods research. Third edition. Los Angeles London New Delhi Singapore Washington DC Melbourne: Sage; 2018

[18] Fetters, M. D., Curry, L. A. & Creswell, J. W. Achieving integration in mixed methods designs-principles and practices. *Health Serv. Res.* **48**(6 Pt 2), 2134–2156 (2013). 24279835 10.1111/1475-6773.12117 PMC4097839

[19] O’Cathain, A., Murphy, E. & Nicholl, J. The quality of mixed methods studies in health services research. *J. Health Serv. Res. Policy* **13**(2), 92–98 (2008). 18416914 10.1258/jhsrp.2007.007074

[20] Couchoud, C. et al. The renal epidemiology and information network (REIN): a new registry for end-stage renal disease in France. *Nephrol. Dial. Transplant* **21**(2), 411–418 (2006). 16234286 10.1093/ndt/gfi198

[21] Audry, B. et al. The new French kidney allocation system for donations after brain death: Rationale, implementation, and evaluation. *Am. J. Transplant* **22**(12), 2855–2868 (2022). 36000787 10.1111/ajt.17180

[22] Launoy, G., Launay, L., Dejardin, O., Bryère, J. & Guillaume, E. European deprivation index: designed to tackle socioeconomic inequalities in cancer in Europe. *Eur. J. Public Health.* **28**(213), cky625 (2018).

[23] Agence de la Biomédecine. Rapport REIN 2021 [Internet]. La plaine, Saint-Denis: 2023 [cited 2023 Nov 24]. Available from: https://www.agence-biomedecine.fr/IMG/pdf/rapport_rein_2021_2023-06-26.pdf

[24] Azur, M. J., Stuart, E. A., Frangakis, C. & Leaf, P. J. Multiple imputation by chained equations: what is it and how does it work?. *Int. J. Methods Psychiatr. Res.* **20**(1), 40–49 (2011). 21499542 10.1002/mpr.329 PMC3074241

[25] Sterne, J. A. C. et al. Multiple imputation for missing data in epidemiological and clinical research: potential and pitfalls. *BMJ* **338**, b2393–b2393 (2009). 19564179 10.1136/bmj.b2393 PMC2714692

[26] Naeem, M., Ozuem, W., Howell, K. & Ranfagni, S. A step-by-step process of thematic analysis to develop a conceptual model in qualitative research. *Int. J. Qual. Methods* **22**, 16094069231205788 (2023).

[27] Tong, A., Sainsbury, P. & Craig, J. Consolidated criteria for reporting qualitative research (COREQ): a 32-item checklist for interviews and focus groups. *Int. J. Qual. Health Care* **19**(6), 349–357 (2007). 17872937 10.1093/intqhc/mzm042

[28] Fetters MD. The Mixed Methods Research Workbook: Activities for Designing, Implementing, and Publishing Projects [Internet]. SAGE Publications, Inc.; 2020 [cited 2024 Aug 13]. Available from: https://methods.sagepub.com/book/the-mixed-methods-research-workbook

[29] Skamagki, G., King, A., Carpenter, C. & Wåhlin, C. The concept of integration in mixed methods research: a step-by-step guide using an example study in physiotherapy. *Physiother. Theory Pract.* **40**(2), 197–204 (2024). 36069530 10.1080/09593985.2022.2120375

[30] Gibbons, A. et al. Patient preferences, knowledge and beliefs about kidney allocation: qualitative findings from the UK-wide ATTOM programme. *BMJ Open* **7**(1), e013896 (2017). 28132010 10.1136/bmjopen-2016-013896 PMC5278279

[31] Jones, E. L., Shakespeare, K., McLaughlin, L. & Noyes, J. Understanding people’s decisions when choosing or declining a kidney transplant: a qualitative evidence synthesis. *BMJ Open* **13**(8), e071348 (2023). 37562929 10.1136/bmjopen-2022-071348 PMC10423837

[32] Rosenthal, M. M. et al. Why take the chance? A qualitative grounded theory study of nocturnal haemodialysis recipients who decline kidney transplantation. *BMJ Open* **6**(5), e011951 (2016). 27194322 10.1136/bmjopen-2016-011951 PMC4874163

[33] Morton, R. L. et al. Patient views about treatment of stage 5 CKD: A qualitative analysis of semistructured interviews. *Am. J. Kidney Dis.* **55**(3), 431–440 (2010). 20116914 10.1053/j.ajkd.2009.11.011

[34] Malik, S. et al. Dialysis decision making, dialysis experiences, and illness perceptions: a qualitative study of pakistani patients receiving maintenance hemodialysis. *Kidney Med.* **4**(11), 100550 (2022). 36353650 10.1016/j.xkme.2022.100550 PMC9637991

[35] DePasquale, N. et al. Selecting renal replacement therapies: what do African American and non-African American patients and their families think others should know? A mixed methods study. *BMC Nephrol.* **14**(1), 9 (2013). 23317336 10.1186/1471-2369-14-9 PMC3565884

[36] Salas, M. A. P. et al. Sex and gender disparity in kidney transplantation: Historical and future perspectives. *Clin. Transplant* **36**(12), e14814 (2022). 36097741 10.1111/ctr.14814 PMC10069947

