# Supplementary material for: A convergent mixed methods to study registration on kidney transplantation waiting list refusal by women and men on dialysis in France
Source: Sci Rep. 2024 Nov 24;14:29106. doi: 10.1038/s41598-024-80775-7 (PMC11586399; doi:10.1038/s41598-024-80775-7)
Supplement: Supplementary file 1 — Supplementary Information. [file 41598_2024_80775_MOESM1_ESM.docx]

**Table s1**: Multivariate logistic model to identify factors associated with registration refusal by patients on dialysis. Analyses included patients who refused kidney transplantation, patients undergoing assessment and patients registered on the waiting list

|  | Odds ratio | | P-value | [95% - | CI] |
| --- | --- | --- | --- | --- | --- |
| Gender | |  |  |  |  |
| Men | | 1 |  |  |  |
| Women | | 1.74 | <0.01 | 1.49 | 2.02 |
| Age group (years) | |  |  |  |  |
| <60 | | 1 |  |  |  |
| ≥60 | | 4.74 | <0.01 | 3.62 | 6.20 |
| EDI | |  |  |  |  |
| Other | | 1 |  |  |  |
| Most deprived | | 0.90 | 0.18 | 0.77 | 1.05 |
| Activity status | |  |  |  |  |
| Active | | 1 |  |  |  |
| Inactive | | 1.47 | 0.11 | 0.90 | 2.38 |
| Hemoglobin (g/dl) | |  |  |  |  |
| <10 | | 1 |  |  |  |
| [10_12] | | 0.93 | 0.41 | 0.79 | 1.09 |
| >12 | | 0.96 | 0.79 | 0.74 | 1.26 |
| Albumin (g/dl) | |  |  |  |  |
| <30 | | 1 |  |  |  |
| ≥30 | | 0.99 | 0.94 | 0.81 | 1.21 |
| BMI (Kg/m²) | |  |  |  |  |
| <18.5 | | 1 |  |  |  |
| [18.5;23[ | | 1.29 | 0.26 | 0.83 | 1.99 |
| [23;25[ | | 1.08 | 0.73 | 0.68 | 1.72 |
| [25;30] | | 0.94 | 0.80 | 0.61 | 1.47 |
| ≥30 | | 0.98 | 0.95 | 0.63 | 1.54 |
| Number of physical disabilities | |  |  |  |  |
| 0 | | 1 |  |  |  |
| ≥1 | | 1.43 | 0.03 | 1.04 | 1.96 |
| Liver disease | |  |  |  |  |
| No | | 1 |  |  |  |
| Yes | | 0.56 | 0.03 | 0.33 | 0.93 |
| First dialysis session autonomous | |  |  |  |  |
| No | | 1 |  |  |  |
| Yes | | 1.59 | <0.01 | 1.22 | 2.08 |
| Number of cardiovascular diseases | |  |  |  |  |
| 0 | | 1 |  |  |  |
| 1 | | 1.43 | <0.01 | 1.19 | 1.72 |
| 2 | | 1.83 | <0.01 | 1.47 | 2.27 |
| ≥3 | | 1.59 | <0.01 | 1.25 | 2.03 |
| Primary kidney disease | |  |  |  |  |
| Polycystic disease | | 1 |  |  |  |
| Diabetes | | 1.44 | 0.05 | 0.99 | 2.10 |
| Glomerulonephritis | | 1.30 | 0.17 | 0.89 | 1.90 |
| Hypertensive and vascular disease | | 1.82 | <0.01 | 1.29 | 2.56 |
| Other/Unknown | | 1.75 | <0.01 | 1.25 | 2.48 |
| Pyelonephritis | | 2.46 | <0.01 | 1.57 | 3.83 |
| Walking impairment | |  |  |  |  |
| Impossible walk | | 1 |  |  |  |
| Walk with assistance | | 0.83 | 0.61 | 0.41 | 1.68 |
| Autonomous | | 0.75 | 0.36 | 0.40 | 1.41 |
| Respiratory insufficiency | |  |  |  |  |
| No | | 1 |  |  |  |
| Yes | | 0.94 | 0.57 | 0.75 | 1.17 |
| Active malignancy | |  |  |  |  |
| No | | 1 |  |  |  |
| Yes | | 0.69 | 0.06 | 0.47 | 1.02 |
| Diabetes | |  |  |  |  |
| No | | 1 |  |  |  |
| Yes | | 1.22 | 0.04 | 1.01 | 1.48 |
| Psychiatric disorder | |  |  |  |  |
| No | | 1 |  |  |  |
| Yes | | 2.23 | <0.01 | 1.38 | 3.60 |
| Ownership of nephrology facility | |  |  |  |  |
| Public not university centre | | 1 |  |  |  |
| Private for profit | | 0.71 | <0.01 | 0.59 | 0.86 |
| Private not for profit | | 0.96 | 0.68 | 0.78 | 1.18 |
| Public university centre | | 0.58 | <0.01 | 0.43 | 0.80 |
| Facility performing kidney transplantation | |  |  |  |  |
| No | | 1 |  |  |  |
| Yes | | 1.16 | 0.38 | 0.83 | 1.62 |
| Emergency dialysis start | |  |  |  |  |
| No | | 1 |  |  |  |
| Yes | | 0.98 | 0.88 | 0.81 | 1.20 |
| First dialysis with catheter | |  |  |  |  |
| No | | 1 |  |  |  |
| Yes | | 1.04 | 0.62 | 0.88 | 1.24 |
| Dialysis modality | |  |  |  |  |
| Hemodialysis | | 1 |  |  |  |
| Peritoneal dialysis | | 0.83 | 0.14 | 0.65 | 1.06 |
